# Prognostic value of [^18^F]fluorodeoxyglucose PET/CT in the new staging system for non-small cell lung cancer

**DOI:** 10.1007/s00330-025-11761-4

**Published:** 2025-06-19

**Authors:** Wonseok Whi, Hyunjong Lee, Sang-Won Um, Hong Kwan Kim, Hong Ryul Pyo, Myung-Ju Ahn, Joon Young Choi

**Affiliations:** 1 Department of Nuclear Medicine, Samsung Medical Center, Sungkyunkwan University School of Medicine, Seoul, Republic of Korea; 2 Division of Pulmonary and Critical Care Medicine, Department of Medicine, Samsung Medical Center, Sungkyunkwan University School of Medicine, Seoul, Republic of Korea; 3 Department of Thoracic and Cardiovascular Surgery, Samsung Medical Center, Sungkyunkwan University School of Medicine, Seoul, Republic of Korea; 4 Department of Radiation Oncology, Samsung Medical Center, Sungkyunkwan University School of Medicine, Seoul, Republic of Korea; 5 Division of Hematology-Oncology, Department of Medicine, Samsung Medical Center, Sungkyunkwan University School of Medicine, Seoul, Republic of Korea

**Keywords:** Non-small cell lung cancer, [^18^F]fluorodeoxyglucose, Positron emission tomography, Staging, Prognosis

## Abstract

**Objective:**

This study aims to explore the prognostic value of primary tumor [^18^F]fluorodeoxyglucose (FDG) uptake in non-small cell lung cancer (NSCLC) patients treated with curative therapy, particularly when considered alongside the new 9th edition of the American Joint Committee on Cancer (AJCC)/Union for International Cancer Control (UICC) staging system.

**Materials and methods:**

A single-center retrospective study analyzed 3070 NSCLC patients who underwent pretherapeutic FDG PET/CT for initial staging. The survival analyses considered clinical variables, disease stage, and the primary tumor’s maximum standardized uptake value (SUVmax). Univariate and multivariate analyses evaluated the prognostic significance of disease stage and SUVmax in predicting overall and disease-free survival. A new staging system incorporating SUVmax is proposed and compared with the conventional staging.

**Results:**

Two thousand nine hundred seventy-two patients (mean age, 64.5 ± 10.1 years; 1888 men) were evaluated. Primary tumor SUVmax was an independent prognostic factor in the univariate and multivariate analyses for overall and disease-free survival, alongside disease stages. Integrating SUVmax into the staging system improved prognostic stratification, especially in intermediate stages (stage IIA vs IIB, hazard ratio [HR] = 1.06, *p* = 0.72 for the conventional stage; HR = 1.27, *p* = 0.04 for the new proposed stage with SUVmax). Survival analyses revealed significant distinctions between reclassified groups based on SUVmax, highlighting its potential for risk assessment refinement.

**Conclusions:**

The primary tumor SUVmax adds prognostic value to the 9th AJCC/UICC staging system for NSCLC. The proposed staging system incorporating SUVmax demonstrates enhanced prognostic accuracy compared with the conventional system.

**Key Points:**

***Question*** *The new NSCLC staging system does not incorporate tumor metabolism, which may enhance prognostic accuracy and improve risk stratification*.

***Findings*** *Primary tumor FDG uptake was an independent prognostic factor for survival in NSCLC. Its integration into staging improved risk stratification*.

***Clinical relevance*** *Primary tumor FDG uptake provides prognostic information in NSCLC. Its incorporation into staging improved risk classification, particularly in intermediate stages, allowing for more precise prognostication based on metabolic activity*.

**Graphical Abstract:**

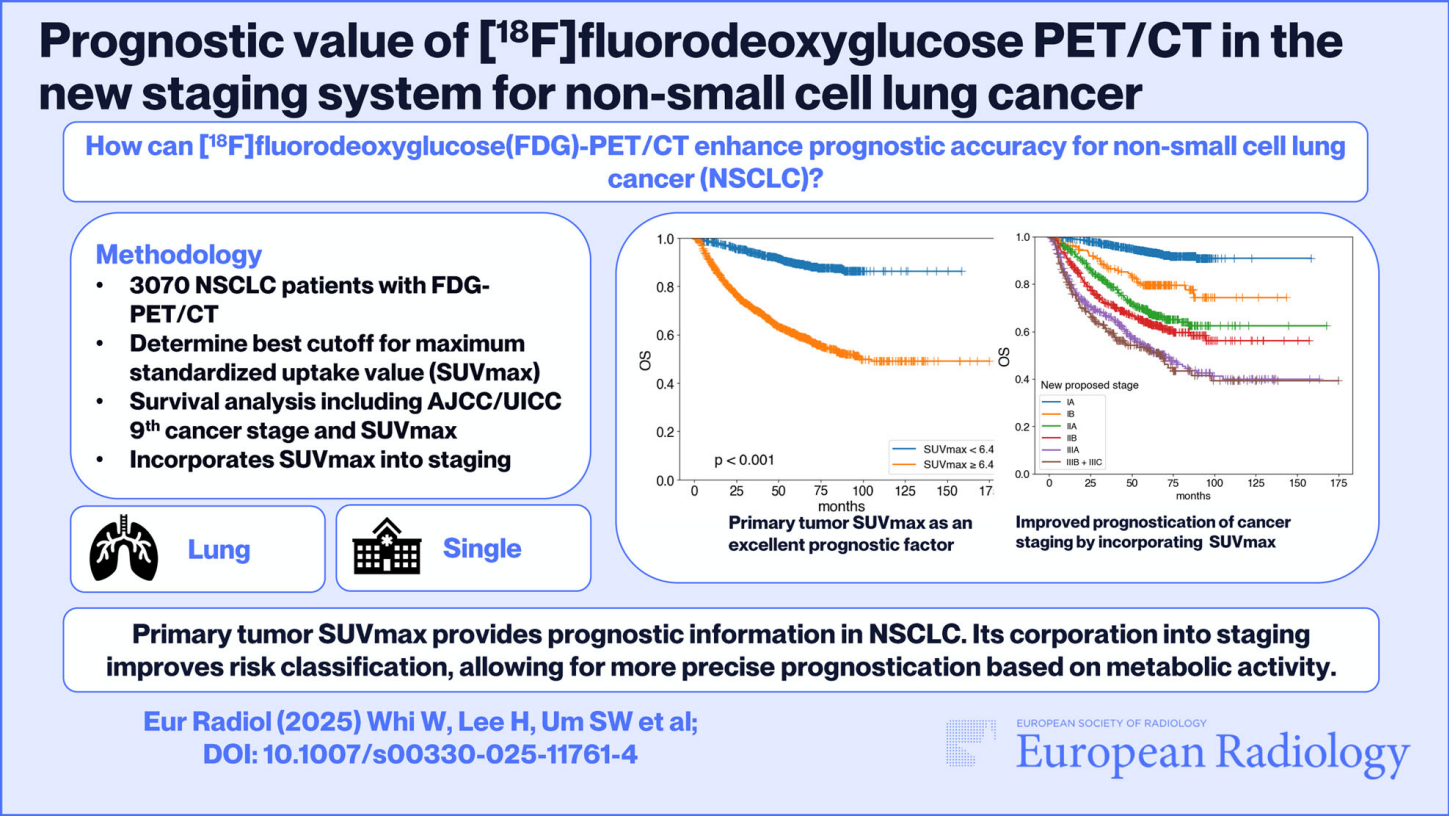

## Introduction

Lung cancer remains one of the leading causes of cancer-related mortality worldwide, with non-small cell lung cancer (NSCLC) accounting for approximately 84% of all lung cancer cases [1]. Accurate staging and prognosis assessment of NSCLC after definitive therapy are crucial for guiding treatment and optimizing patient care. [^18^F]Fluorodeoxyglucose (FDG) positron emission tomography (PET)/computed tomography (CT) is an essential tool in the initial staging and prognostication of NSCLC [2, 3]. In addition to detecting metastatic lesions, PET/CT provides metabolic parameters such as the maximum standardized uptake value (SUVmax), which reflects tumor glucose metabolism. Numerous studies have demonstrated that higher SUVmax values are associated with poorer prognoses in various cancers, including NSCLC [4, 5]. Notably, a previous study has reported that incorporating SUVmax into the current staging system may enhance prognostic accuracy [6].

The cancer staging system developed and suggested by the American Joint Committee on Cancer (AJCC)/Union for International Cancer Control (UICC) is the most widely adopted framework for assessing the extent and progression of malignancies, including NSCLC. This system provides a standardized approach for classifying a tumor’s primary size and extent (T), lymph node involvement (N), and presence of metastases (M) to guide clinical decision-making and prognostication. In 2024, the 9th edition of the AJCC/UICC staging system for NSCLC was proposed [7, 8]. A notable change between the 8th and 9th editions of the system is the further subdivision of the N2 stage into N2a and N2b based on the number of involved lymph node stations, which were previously classified as N2 metastasis. This modification has led to the reclassification of stages II and III and their respective substages. Additionally, the 9th edition introduced a distinction within the M1c category, further dividing it into M1c1 and M1c2 based on the number of organs involved in metastases. The criteria for the T stage have remained consistent. In a survival analysis, the staging system based on the 9th edition showed enhanced prognostic stratification compared with the 8th edition [7]. However, no prior studies have investigated the independent prognostic significance of FDG PET/CT parameters, such as SUVmax, within the framework of the 9th edition AJCC/UICC staging system for lung cancer.

In this retrospective study, we investigated the prognostic value of primary tumor FDG uptake shown as SUVmax according to the 8th and 9th editions of the AJCC/UICC staging system in a large cohort of patients with NSCLC. Additionally, we explored whether incorporating SUVmax into the 9th edition AJCC/UICC staging could further enhance prognostic accuracy.

## Materials and methods

### Subjects

Our institutional review board (IRB) approved this retrospective study and waived the requirement for informed consent (IRB No. 2024-12-095). We retrospectively enrolled 3,070 consecutive patients who underwent FDG PET/CT examination for initial staging of NSCLC and subsequent definitive therapies between January 2007 and December 2016. Among them, 13 patients with a pathologic stage of T0 or Tis and 35 patients with distant metastasis (M1) were excluded. Fourteen patients without properly documented pathologic information were also excluded, along with eight patients whose data were inadequate for clinical staging. Finally, 28 patients without an adequately documented FDG PET/CT acquisition protocol were excluded. Therefore, data from 2972 patients were analyzed in this study.

All primary lung cancer lesions were pathologically confirmed using one of the following methods: surgical specimens for patients who underwent surgical resection, or biopsy and cytology for patients who did not undergo surgery. For all included NSCLC patients, chest X-ray, contrast-enhanced chest CT, FDG PET/CT, and bronchoscopy were performed as the standard protocol for initial staging of the disease. Additional radiologic tests, including endobronchial ultrasound-guided biopsy, brain magnetic resonance imaging (MRI), contrast-enhanced abdominal CT, contrast-enhanced adrenal CT, and liver MRI, were performed as clinically indicated based on findings from prior examinations that warranted further evaluation of specific regions.

### FDG PET/CT acquisition and analysis

All patients fasted for at least 6 h, and the blood glucose concentration was restricted to < 200 mg/dL at the time of FDG injection. Imaging was performed using a Discovery LS (GE Healthcare) or Discovery STE (GE Healthcare) after 60 min of FDG distribution (5 MBq/kg or 13.5 μCi/kg). Continuous spiral CT scans were performed using eight-slice helical CT (140 keV, 40–120 mA, Discovery LS) or 16-slice helical CT (140 keV, 30–170 mA, Discovery STE). Emission scans were obtained from head to thigh, at 4 min per frame in 2D mode (Discovery LS) or 2.5 min per frame in 3D mode (Discovery STE). PET images were reconstructed with CT for attenuation correction using the ordered-subsets expectation maximization algorithm with 28 subsets and two iterations (matrix 128 × 128, voxel size 4.3 × 4.3 × 3.9 mm, Discovery LS) or the ordered-subsets expectation maximization algorithm with 20 subsets and two iterations (matrix 128×128, voxel size 3.9 × 3.9 × 3.3 mm, Discovery STE). The SUV was calculated with adjustment for the administered FDG dose and body weight.

All images were interpreted by two or more experienced nuclear medicine physicians, and at least one had more than 10 years of experience in reading FDG PET/CT results. Image interpretation was based on visual inspection with semiquantitative analyses. Using volume viewer software on a GE Advantage Workstation version 4.7, the primary tumor SUVmax was measured in a spherical volume of interest, as no other volumetric parameters were utilized in this study.

### Collection of clinical variables

Clinical information (sex, age, and histological grade of the primary tumor) was obtained by reviewing electronic medical records. We assigned a pathologic stage for each patient undergoing initial curative surgery and a clinical stage for those treated with definitive radiotherapy or curative surgery preceded by neoadjuvant concurrent chemoradiotherapy (CCRT). Pathology reports acquired from surgery were reviewed to determine the pathologic stage. Radiologic reports of FDG PET/CT and chest CT scans were reviewed for clinical staging. The size of the primary tumor was determined based on the CT scan. The clinical nodal stage was evaluated by considering a lymph node station as positive if any of the FDG PET/CT, chest CT, or fine needle aspiration biopsy (if performed) results showed positivity. Both staging procedures were performed based on the 8th and 9th editions of the AJCC/UICC staging system.

For 2043 surgically treated patients, hilar and mediastinal lymphadenectomy was performed in 1666 (81.5%) cases. Among them, systematic lymph node dissection was conducted in 1550 (93.0%) cases, while lymph node sampling was performed in 116 (7.0%) cases based on intraoperative findings and surgical judgment. Adjuvant therapy after surgery was performed based on the patient's situation and the physician's decision. After surgery, all patients were monitored regularly to obtain accurate information about recurrence. Patients were scheduled for routine follow-up every 3–6 months for the first three years after curative-intent therapy and then every six months thereafter. Every follow-up evaluation included a physical examination, laboratory tests, biochemical screening, chest X-ray, and chest CT scan. FDG PET/CT or other relevant tests were also performed if clinically indicated. Recurrence or metastasis was considered when there was an abnormal finding on serial imaging studies or pathologically confirmed malignancy. The events for survival analysis were defined as recurrence or metastasis and death from any cause. The overall survival (OS) and disease-free survival (DFS) durations were defined as the intervals from the date of histological diagnosis to the date of death, disease recurrence, or the last follow-up.

### Statistical analysis

Age was divided into three ranges as a discrete scale according to tertile points for the log-rank tests and multivariate analyses. SUVmax values were harmonized to remove batch effects due to varying PET/CT instruments, using the package *neuroCombat* in R. Harmonized SUVmax values were divided into two groups as a discrete scale (high and low) at the value that best discriminated the OS in all subjects; in other words, the cutoff was statistically determined by maximizing the rank-based test statistic for OS. An area under the receiver operating characteristic curve (AUC) analysis was additionally performed to evaluate the discriminatory performance of this cutoff. Cutoff values were applied not only for the log-rank tests and multivariate analyses but also as suggestions for a reference value in the new staging system.

Sex, age (as both continuous and discrete variables), adjuvant therapy, histological grade of the primary tumor, pathological/clinical T/N stages, stage (e.g., IA and IB) based on 8th and 9th editions of the AJCC/UICC staging system, and SUVmax (as both discrete and continuous scales) were used in the univariate survival analyses. Due to the small sample size of Stage IA1 and the complexity of the study design, Stage IA subgroups (IA1, IA2, and IA3) were analyzed as a combined Stage IA group.

Both OS and DFS were endpoints of the analyses. The Cox proportional hazards model was used to evaluate each variable’s prognostic power. Hazard ratios (HRs) and 95% confidence intervals (CIs) were estimated. Log-rank statistics were obtained using the Kaplan–Meier method. Variables significant in the univariate survival analyses (log-rank statistics with a *p*-value less than 0.05) were included in the multivariate survival analyses. Variables with collinearity were excluded.

Patients in each 9th-edition staging group were divided into two groups based on the reference values for SUVmax. HRs and 95% CIs based on OS were estimated, and log-rank statistics were obtained using the Kaplan–Meier method. Subgroup members were upstaged or downstaged to new groups as needed based on the HRs, with some remaining in their original group. Pair-wise Kaplan–Meier analyses were conducted in the newly classified groups. All statistical analyses were performed using Pandas software version 2.2 (NumFOCUS) and R software (v.4.0.4, R Foundation for Statistical Computing). *p*-values lower than 0.05 were considered statistically significant.

## Results

### Demographic data

**Fig. 1:**
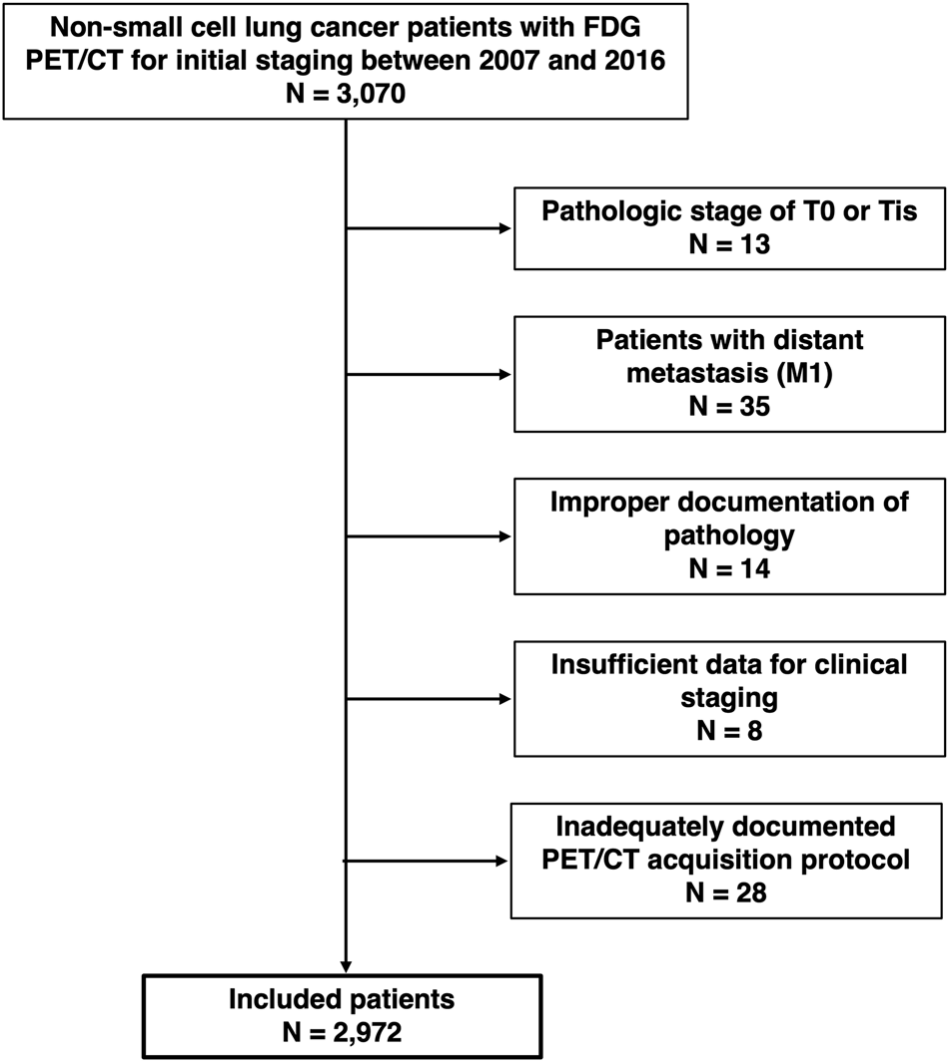
Inclusion and exclusion criteria. This study retrospectively enrolled 3070 consecutive patients with FDG PET/CT results from an initial staging of NSCLC and subsequent definitive therapies between January 2007 and December 2016. Among them, patients with a pathologic stage of T0 or Tis, with distant metastasis (M1), without properly documented pathologic information, without data adequate for clinical staging, or without an adequately documented FDG PET/CT acquisition protocol were excluded. After exclusions, data for 2972 patients were analyzed in this study

A total of 2972 patients were included in the analysis (Fig. 1). The clinical characteristics and demographics of the subjects are described in Table 1. Of the total patients, 63.5% were male. The median age of the subjects was 65 years, and the median SUVmax was 6.6. The cutoff value for SUVmax was determined to be 6.4 (AUC = 0.73, *c*-index = 0.68, sensitivity = 0.58, specificity = 0.79), which was identified as optimal using both rank-based and AUC-based analyses. Stage IA was the most common group according to both the 8th and 9th edition staging criteria, and stage IIIA was the most common clinical substage according to both the 8th and 9th edition criteria. Because the number of subjects with stage IIIC (the criterion for which is unchanged in the 9th edition) was small (*n* = 35), those patients were combined with the IIIB group for analysis.

**Table 1:** Demographic and clinical characteristics of the included NSCLC patients

Variable	Number (%)
Age at diagnosis, range (median)	21–97 (65)
Younger (< 61)	1019 (34.3%)
Intermediate (61–69)	949 (31.9%)
Older (> 69)	1004 (33.8%)
Sex	
Male	1888 (63.5%)
Female	1084 (36.5%)
SUVmax, median	6.6
< 6.4	1466 (49.3%)
≥ 6.4	1506 (50.7%)
Cell type	
Adenocarcinoma	2039 (68.6%)
Non-adenocarcinoma	933 (31.4%)
Treatment modality	
Initial surgery ± adjuvant chemotherapy	2043 (68.7%)
Definitive radiotherapy ± adjuvant chemotherapy	482 (16.2%)
Neoadjuvant chemoradiotherapy + surgery	447 (15.0%)
T stage	
T1a	127 (4.3%)
T1b	586 (19.7%)
T1c	764 (25.7%)
T2a	637 (21.4%)
T2b	287 (9.6%)
T3	394 (13.3%)
T4	177 (6.0%)
8th N stage	
N0	1986 (66.8%)
N1	251 (8.4%)
N2	710 (23.9%)
N3	25 (0.8%)
9th N stage	
N0	1986 (66.8%)
N1	251 (8.4%)
N2a	471 (15.8%)
N2b	239 (8.0%)
N3	25 (0.8%)
8th stage group	
IA	1193 (40.1%)
IB	387 (13.0%)
IIA	122 (4.1%)
IIB	372 (12.5%)
IIIA	670 (22.5%)
IIIB	193 (6.5%)
IIIC	35 (1.2%)
9th stage group	
IA	1193 (40.1%)
IB	387 (13.0%)
IIA	198 (6.7%)
IIB	440 (14.8%)
IIIA	510 (17.2%)
IIIB	209 (7.0%)
IIIC	35 (1.2%)
Instrument	
Discovery LS	736 (24.8%)
Discovery STE	2236 (75.2%)

*SUVmax* maximum standardized uptake value

### Survival analysis data

In univariate survival analysis, age, sex, treatment modality, cell type, T stage, N stage (8th/9th), stage group (8th/9th), and SUVmax as both a continuous and discrete scale were significant prognostic factors for both OS and DFS (Table 2).

**Table 2:** Univariate Cox regression analyses and log-rank analyses of survival in NSCLC patients

Variable	Categories	OS			DFS		
		HR (95% CI)	*p*	Log-rank	HR (95% CI)	*p*	Log-rank
Age	< 61			< 0.001			< 0.001
	61–69	2.03 (1.64–2.51)	< 0.001		1.22 (1.05–1.42)	0.01	
	> 69	3.90 (3.20–4.76)	< 0.001		1.80 (1.56–2.07)	< 0.001	
Age (cont.)		1.06 (1.05–1.07)	< 0.001	< 0.001	1.02 (1.02–1.03)	< 0.001	< 0.001
Sex	Female			< 0.001			< 0.001
	Male	2.56 (2.14–3.06)	< 0.001		1.66 (1.46–1.89)	< 0.001	
Treatment	Surgery^a^			< 0.001			< 0.001
	Definitive RT^a^	5.98 (5.05–7.10)	< 0.001		3.12 (2.69–3.63)	< 0.001	
	Neoadjuvant CCRT^b^	3.18 (2.64–3.84)	< 0.001		3.69 (3.21–4.24)	< 0.001	
Cell type	Adenocarcinoma			< 0.001			< 0.001
	Non-adenocarcinoma	2.95 (2.55–3.42)	< 0.001		1.71 (1.52–1.93)	< 0.001	
T stage	T1a			< 0.001			< 0.001
	T1b	1.78 (0.89–3.56)	0.10		1.76 (1.03–3.02)	0.04	
	T1c	2.49 (1.26–4.90)	0.008		3.07 (1.82–5.18)	< 0.001	
	T2a	4.30 (2.20–8.42)	< 0.001		5.10 (3.05–8.58)	< 0.001	
	T2b	7.43 (3.37–14.66)	< 0.001		7.23 (4.26–12.28)	< 0.001	
	T3	9.04 (4.62–17.67)	< 0.001		7.90 (4.68–13.33)	< 0.001	
	T4	10.97 (5.51–21.80)	< 0.001		8.86 (5.15–15.24)	< 0.001	
8th N stage	N0			< 0.001			< 0.001
	N1	2.17 (1.71–2.76)	< 0.001		2.55 (2.10–3.10)	< 0.001	
	N2	2.66 (2.27–3.11)	< 0.001		3.97 (3.50–4.50)	< 0.001	
	N3	7.48 (4.53–12.36)	< 0.001		6.95 (4.39–11.00)	< 0.001	
9th N stage	N0			< 0.001			< 0.001
	N1	2.17 (1.71–2.76)	< 0.001		2.56 (2.10–3.11)	< 0.001	
	N2a	2.57 (2.14–3.08)	< 0.001		3.84 (3.33–4.43)	< 0.001	
	N2b	2.87 (2.29–3.59)	< 0.001		4.26 (3.58–5.08)	< 0.001	
	N3	7.49 (4.53–12.37)	< 0.001		6.95 (4.39–11.01)	< 0.001	
8th stage group	IA			< 0.001			< 0.001
	IB	2.50 (1.89–3.30)	< 0.001		2.54 (2.04–3.17)	< 0.001	
	IIA	4.28 (3.02–6.07)	< 0.001		3.70 (2.74–4.98)	< 0.001	
	IIB	3.91 (3.03–5.05)	< 0.001		3.69 (2.99–4.54)	< 0.001	
	IIIA	4.75 (3.80–5.92)	< 0.001		6.48 (5.45–7.69)	< 0.001	
	IIIB + IIIC^c^	8.88 (6.88–11.46)	< 0.001		8.75 (7.08–10.82)	< 0.001	
9th stage group	IA			< 0.001			< 0.001
	IB	2.50 (1.89–3.30)	< 0.001		2.54 (2.04–3.17)	< 0.001	
	IIA	3.64 (2.67–4.96)	< 0.001		3.64 (2.83–4.68)	< 0.001	
	IIB	3.84 (3.00–4.91)	< 0.001		4.33 (3.57–5.26)	< 0.001	
	IIIA	5.80 (4.63–7.26)	< 0.001		6.73 (5.62–8.06)	< 0.001	
	IIIB + IIIC^c^	7.18 (5.54–9.31)	< 0.001		8.32 (6.76–10.25)	< 0.001	
SUVmax (categorized)	< 6.4			< 0.001			< 0.001
	≥ 6.4	4.74 (3.97–5.67)	< 0.001		3.87 (3.39–4.42)	< 0.001	
SUVmax (continuous)		1.09 (1.08–1.10)	< 0.001	< 0.001	1.08 (1.07–1.08)	< 0.001	< 0.001

*HR* hazard ratio, *CI* confidence interval, *RT* radiotherapy, *CCRT* concurrent chemoradiotherapy, *SUVmax* maximum standardized uptake value

^a^ With or without subsequent adjuvant chemotherapy

^b^ Followed by surgery

^c^ Due to the small sample size of stage group IIIC, it was combined into the IIIB group

**Fig. 2:**
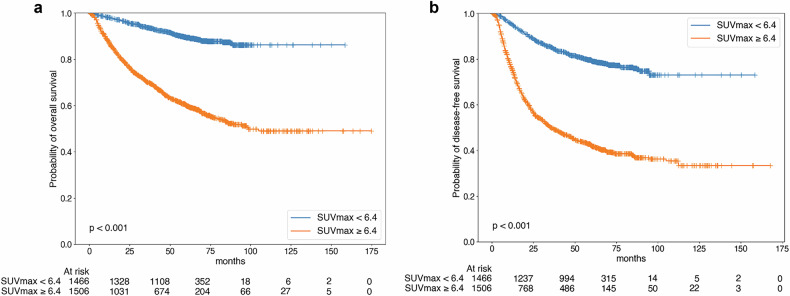
Kaplan–Meier survival curves according to SUVmax. SUVmax discriminated by the best cutoff value for discriminating the prognosis of OS was a significant prognostic factor in both overall (**a**) and DFS (**b**)

**Fig. 3:**
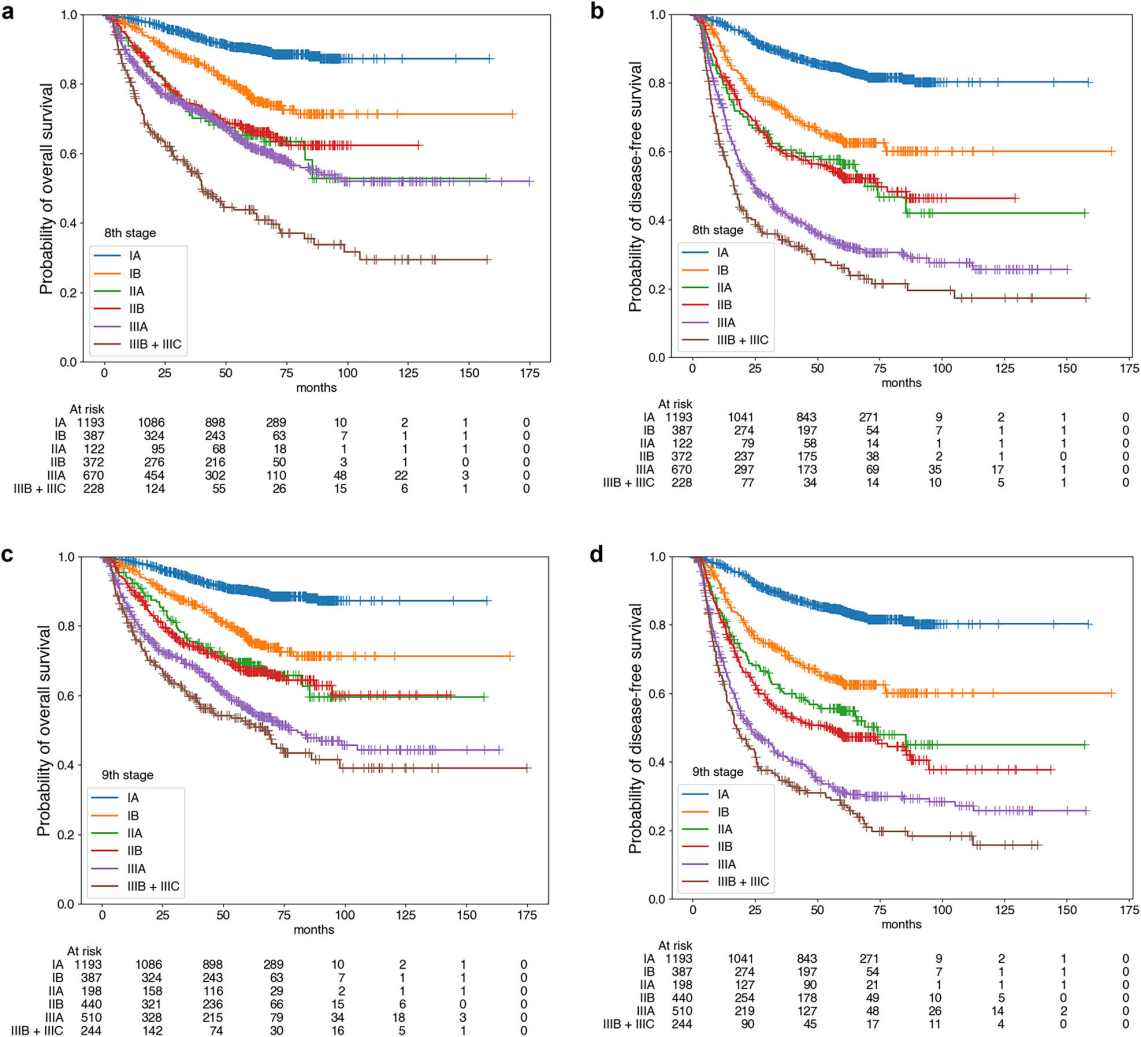
Survival curves for each prognostic stage group suggested by the AJCC/UICC staging guidelines, 8th and 9th editions. For the 8th edition, the prognostic stage group was a significant prognostic factor for both overall (**a**) and disease-free (**b**) survival. The result was the same for the stage groups in the 9th edition for overall (**c**) and disease-free (**d**) survival

Due to multicollinearity issues, the multivariate survival analysis was performed for each PET parameter and staging system. In the multivariate survival analysis, age, sex, treatment modality (definitive radiotherapy group vs initial surgery group), stage group (8th/9th), and SUVmax were significant prognostic factors for OS, and treatment modality (neoadjuvant CCRT + surgery group vs initial surgery group), stage group (8th/9th), and SUVmax were significant prognostic factors for DFS (Supplementary Tables 1 and 2). Survival curves according to SUVmax and the 8th/9th edition stage groups are displayed in Figs. 2 and 3.

### Proposing a new staging system including SUVmax

Subgroup analyses were performed to explore the role of SUVmax in each stage and adjust the staging system to achieve more accurate prognoses. HRs for OS were calculated in subgroups classified according to each 9th edition cancer stage and SUVmax group (Supplementary Table 3). Our proposed new staging system was designed based on those results. Stage IB subjects with low SUVmax were downstaged to a new stage IA. Stage IIA and IIB subjects with low SUVmax were downstaged to a new stage IB. Stage IA and IB subjects with high SUVmax were upstaged to stage IIA. Stage IIA patients with high SUVmax were upstaged, and stage IIIA patients with low SUVmax were downstaged to the new stage IIB. These changes are summarized in Table 3.

**Table 3:** The new proposed staging system is based on the AJCC/UICC 9th edition stage group and SUVmax

AJCC/UICC 9th ed. stage group	SUVmax^a^	New proposed stage group
IA	Low	IA
IB	Low	
IIA	Low	IB
IIB	Low	
IA	High	IIA
IB	High	
IIA	High	IIB
IIB	High	
IIIA	Low	
IIIA	High	IIIA
IIIB		IIIB
IIIC		

*AJCC* American Joint Committee on Cancer, *UICC* Union for International Cancer Control, *SUVmax* maximum standardized uptake value

^a^ Categorized by a cutoff value of 6.4, as determined to discriminate the OS best

**Fig. 4:**
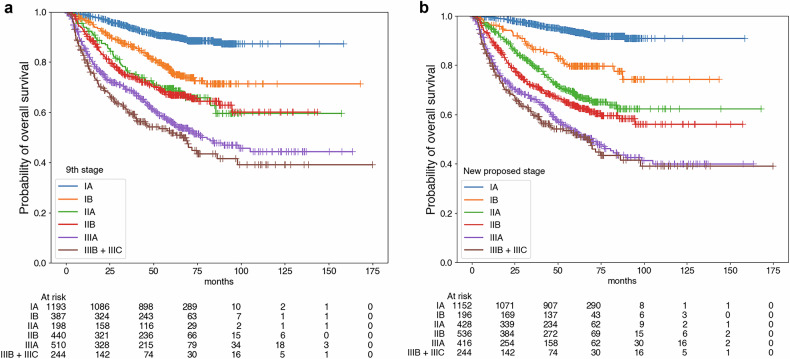
Survival curves according to AJCC/UICC 9th edition cancer stages and the new proposed stages. Compared with survival curves based on the 9th edition stage groups (**a**), subjects in stages IIA and IIB in the new proposed staging system showed significant differences in OS when SUVmax was incorporated into the staging system (**b**). However, the new staging system still did not discriminate against subjects in stages IIIA and IIIB

In the conventional staging system, OS did not differ significantly between subjects in stages IIA/IIB (HR =1.06, *p* = 0.72) and IIIA/IIIB (HR = 1.22, *p* = 0.08). In the new staging system, subjects in stages IIA and IIB did differ significantly in OS (HR = 1.27, *p* = 0.04), though subjects in stages IIIA and IIIB were still not discriminated (HR = 1.10, *p* = 0.42) (Table 4). Compared with survival curves based on the 9th edition stage groups, those based on our proposed staging system better discriminated prognoses between stages IIA and IIB (Fig. 4).

**Table 4:** Univariate Cox regression analyses (pairwise) of OS according to the conventional system and the newly proposed staging system that considers the SUVmax of the primary NSCLC lesion

	Conventional staging (9th ed.)		New proposed staging	
	HR (95% CI)	*p*	HR (95% CI)	*p*
Stage IA vs IB	2.54 (1.92–3.36)	< 0.001	3.20 (2.17–4.73)	< 0.001
Stage IB vs IIA	1.47 (1.06–2.03)	0.02	1.74 (1.21–2.50)	0.003
Stage IIA vs IIB	1.06 (0.78–1.43)	0.72	1.27 (1.01–1.58)	0.04
Stage IIB vs IIIA	1.50 (1.21–1.86)	< 0.001	1.45 (1.18–1.77)	< 0.001
Stage IIIA vs IIIB^a^	1.22 (0.97–1.54)	0.08	1.10 (0.87–1.39)	0.42

*HR* hazard ratio, *CI* confidence interval

^a^ Incorporates stage IIIC from the conventional system

## Discussion

In this study, we identified the SUVmax of primary tumors from FDG PET/CT scans as a significant prognostic factor for patients with NSCLC, independent of the 8th and 9th edition AJCC/UICC tumor, node, metastasis (TNM) staging systems. Both our univariate and multivariate analyses demonstrated the prognostic power of SUVmax, highlighting its ability to provide additional stratification within individual stage groups. Incorporating SUVmax into the staging system improved prognostic discrimination compared with the conventional staging methods alone. Thus, a staging system integrating primary tumor FDG uptake could be a refined tool for risk assessment.

The SUVmax of the primary tumor, the most widely used semi-quantitative parameter from FDG PET/CT, reflects tumor glucose metabolism and serves as an indicator of biological aggressiveness [9]. Numerous studies have emphasized its utility, consistently showing that higher SUVmax values are linked to poorer outcomes across various cancers, including NSCLC [10–13]. One of the key advantages of this metric is its ease of measurement and high reproducibility, making it particularly suitable for routine clinical application. As a result, SUVmax is widely adopted in clinical practice as a versatile and practical parameter.

FDG PET/CT plays a pivotal role in both the diagnostic and prognostic landscapes of NSCLC [14]. Previous studies have demonstrated the prognostic relevance of SUVmax in NSCLC. For example, elevated SUVmax values have been associated with poor survival [4, 11] and high recurrence rates [5, 12]. However, most prior research has been limited by small cohorts or not based on recent staging revisions. For example, Takeda et al recruited 95 patients who received stereotactic body radiotherapy [5], Imamura et al enrolled 62 NSCLC patients receiving chemotherapy [15], and Yoo et al used 80 patients without lymph node metastasis [16]. Some meta-analyses included multiple studies and a larger number of patients: Berghsmans et al enrolled 13 studies and 1474 NSCLC patients [4], and Paesman et al included 2637 patients from 21 studies [11]. To our knowledge, the present study is the first single-center study to analyze the prognostic significance of SUVmax in a cohort of this scale under the framework of the updated 9th edition AJCC/UICC staging system.

In current practice, the management of NSCLC is primarily guided by the TNM staging system, with treatment options ranging from surgical resection for early-stage disease to systemic therapy for advanced stages [17, 18]. However, our study suggests that incorporating SUVmax from FDG PET/CT scans could refine this approach, particularly for patients with stage II or lower NSCLC. Patients with high SUVmax, even within the early stages, might represent a high-risk group that could benefit from more aggressive treatment strategies or closer surveillance. For instance, stage II patients with high SUVmax might be considered for more intensive adjuvant therapy or shorter follow-up intervals, similar to the approach for higher-stage disease. Conversely, patients with low SUVmax values might be candidates for less intensive follow-up regimens. This nuanced approach could lead to more personalized treatment plans, potentially improving outcomes while avoiding overtreatment in lower-risk patients. Further prospective studies are needed to validate the clinical impact of incorporating SUVmax into treatment algorithms, but our findings suggest that it is a promising avenue for enhancing the precision of NSCLC management.

Despite its potential, this study has limitations. First, pathological staging was available only for surgically treated patients, and significant delays between clinical and pathological staging may have introduced bias. However, a subgroup analysis restricted to patients with pathological staging showed consistent results, supporting the robustness of our findings (Supplementary Tables 7 and 8). Second, as a retrospective study, treatment strategies were based on the 6th and 7th editions of the AJCC/UICC staging systems, potentially introducing heterogeneity and not accounting for recent advancements in treatment modalities and concepts, which have significantly improved survival outcomes in NSCLC patients. To address this concern, we performed an additional subgroup analysis limited to patients initially staged and managed according to the 7th edition, which demonstrated consistent prognostic results (Supplementary Tables 9 and 10). Third, as a retrospective study, detailed nodal information required for rigorous pathological nodal staging according to the latest 9th TNM edition was not fully available due to practical considerations, given the invasive nature and associated risks of procedures. Moreover, quantitative nodal FDG uptake parameters from PET/CT, which might have provided additional prognostic value, were not systematically analyzed, warranting further prospective studies. Fourth, volume-based PET parameters, such as metabolic tumor volume or total lesion glycolysis, which are generally considered superior to SUVmax for prognostic prediction [19, 20], were not included in this study. However, SUVmax is more clinically applicable than volume-based parameters due to its simplicity and ease of measurement, and the favorable results of this study demonstrate its value. Fifth, while our proposed staging system demonstrated improved prognostic stratification, the modified clinical decisions by up- or down-staging regarding treatment are not reflected in the prognosis. Prospective validation studies are required to clarify its role in guiding treatment decisions. Finally, as a single-center study, our proposed staging system incorporating SUVmax requires external validation through collaboration with other high-volume lung cancer centers to confirm reproducibility and generalizability.

In conclusion, the primary tumor FDG uptake, shown as SUVmax on PET/CT scans, was identified as an independent prognostic factor in NSCLC patients receiving curative treatment, along with the stage group according to both the 8th and 9th editions of the AJCC/UICC staging systems. The newly proposed staging system incorporating SUVmax demonstrates the potential to discriminate prognoses better than the conventional staging approach.

## Supplementary information

ELECTRONIC SUPPLEMENTARY MATERIAL

## Abbreviations

AJCC: American Joint Committee on Cancer
CCRT: Concurrent chemoradiotherapy
CI: Confidence interval
DFS: Disease-free survival
FDG: Fluorodeoxyglucose
HR: Hazard ratio
IRB: Institutional Review Board
MRI: Magnetic resonance imaging
NSCLC: Non-small cell lung cancer
OS: Overall survival
PET/CT: Positron emission tomography/computed tomography
SUVmax: Maximum standardized uptake value
TNM: Tumor, node, metastasis
UICC: Union for International Cancer Control

## References

[1] Ganti AK, Klein AB, Cotarla I, Seal B, Chou E (2021) Update of incidence, prevalence, survival, and initial treatment in patients with non-small cell lung cancer in the US. JAMA Oncol 7:1824–1832 34673888 10.1001/jamaoncol.2021.4932 PMC8532041

[2] Shim SS, Lee KS, Kim B-T et al (2005) Non-small cell lung cancer: prospective comparison of integrated FDG PET/CT and CT alone for preoperative staging. Radiology 236:1011–1019 16014441 10.1148/radiol.2363041310

[3] Kandathil A, Kay FU, Butt YM, Wachsmann JW, Subramaniam RM (2018) Role of FDG PET/CT in the eighth edition of TNM staging of non-small cell lung cancer. Radiographics 38:2134–2149 30422775 10.1148/rg.2018180060

[4] Berghmans T, Dusart M, Paesmans M et al (2008) Primary tumor standardized uptake value (SUVmax) measured on fluorodeoxyglucose positron emission tomography (FDG-PET) is of prognostic value for survival in non-small cell lung cancer (NSCLC): a systematic review and meta-analysis (MA) by the European Lung Cancer Working Party for the IASLC Lung Cancer Staging Project. J Thorac Oncol 3:6–12 18166834 10.1097/JTO.0b013e31815e6d6b

[5] Takeda A, Yokosuka N, Ohashi T et al (2011) The maximum standardized uptake value (SUVmax) on FDG-PET is a strong predictor of local recurrence for localized non-small-cell lung cancer after stereotactic body radiotherapy (SBRT). Radiother Oncol 101:291–297 21889224 10.1016/j.radonc.2011.08.008

[6] Umakoshi H, Iwano S, Yokoi K et al (2018) FDG PET/CT overcomes discordance between clinical and pathologic TNM classification of small-size primary lung cancer: influence on postoperative prognosis. Clin Lung Cancer 19:e37–e45 28666761 10.1016/j.cllc.2017.05.021

[7] Rami-Porta R, Nishimura KK, Giroux DJ et al (2024) The International Association for the Study of Lung Cancer Lung Cancer Staging project: proposals for revision of the TNM stage groups in the forthcoming (Ninth) edition of the TNM classification for lung cancer. J Thorac Oncol 19:1007–1027 10.1016/j.jtho.2024.02.011 38447919

[8] Detterbeck FC, Woodard GA, Bader AS et al (2024) The proposed 9th edition TNM classification of lung cancer. Chest 166:882–895 10.1016/j.chest.2024.05.026 38885896

[9] Lucignani G, Paganelli G, Bombardieri E (2004) The use of standardized uptake values for assessing FDG uptake with PET in oncology: a clinical perspective. Nucl Med Commun 25:651–656 15208491 10.1097/01.mnm.0000134329.30912.49

[10] Cerfolio RJ, Bryant AS, Ohja B, Bartolucci AA (2005) The maximum standardized uptake values on positron emission tomography of a non-small cell lung cancer predict stage, recurrence, and survival. J Thorac Cardiovasc Surg 130:151–159 15999056 10.1016/j.jtcvs.2004.11.007

[11] Paesmans M, Berghmans T, Dusart M et al (2010) Primary tumor standardized uptake value measured on fluorodeoxyglucose positron emission tomography is of prognostic value for survival in non-small cell lung cancer: update of a systematic review and meta-analysis by the European Lung Cancer Working Party for the International Association for the Study of Lung Cancer Staging Project. J Thorac Oncol 5:612–619 20234323 10.1097/JTO.0b013e3181d0a4f5

[12] Cerfolio RJ, Bryant AS (2006) Maximum standardized uptake values on positron emission tomography of esophageal cancer predicts stage, tumor biology, and survival. Ann Thorac Surg 82:391–395 16863735 10.1016/j.athoracsur.2006.03.045

[13] Cacicedo J, Fernandez I, Del Hoyo O et al (2017) Prognostic value of maximum standardized uptake value measured by pretreatment 18F-FDG PET/CT in locally advanced head and neck squamous cell carcinoma. Clin Transl Oncol 19:1337–1349 28540535 10.1007/s12094-017-1674-6

[14] Grootjans W, de Geus-Oei L-F, Troost EG, Visser EP, Oyen WJ, Bussink J (2015) PET in the management of locally advanced and metastatic NSCLC. Nat Rev Clin Oncol 12:395–407 25917254 10.1038/nrclinonc.2015.75

[15] Imamura Y, Azuma K, Kurata S et al (2011) Prognostic value of SUVmax measurements obtained by FDG-PET in patients with non-small cell lung cancer receiving chemotherapy. Lung Cancer 71:49–54 20430470 10.1016/j.lungcan.2010.04.004

[16] Yoo IR, Chung SK, Park HL et al (2014) Prognostic value of SUVmax and metabolic tumor volume on 18F-FDG PET/CT in early stage non-small cell lung cancer patients without LN metastasis. Biomed Mater Eng 24:3091–3103 25227018 10.3233/BME-141131

[17] Tsim S, O’dowd C, Milroy R, Davidson S (2010) Staging of non-small cell lung cancer (NSCLC): a review. Respir Med 104:1767–1774 20833010 10.1016/j.rmed.2010.08.005

[18] Riely GJ, Wood DE, Ettinger DS et al (2024) Non-small cell lung cancer, version 4.2024, NCCN clinical practice guidelines in oncology. J Natl Compr Cancer Netw 22:249–274 10.6004/jnccn.2204.0023 38754467

[19] Moon SH, Hyun SH, Choi JY (2013) Prognostic significance of volume-based PET parameters in cancer patients. Korean J Radiol 14:1–12 23323025 10.3348/kjr.2013.14.1.1 PMC3542291

[20] Hyun SH, Ahn HK, Kim H et al (2014) Volume-based assessment by 18 F-FDG PET/CT predicts survival in patients with stage III non-small-cell lung cancer. Eur J Nucl Med Mol Imaging 41:50–58 23948859 10.1007/s00259-013-2530-8

