# Supplementary material for: Prognostic value of [18F]fluorodeoxyglucose PET/CT in the new staging system for non-small cell lung cancer
Source: Eur Radiol. 2025 Jun 19;35(12):7669–79. doi: 10.1007/s00330-025-11761-4 (PMC12634719; doi:10.1007/s00330-025-11761-4)
Supplement: Supplementary file 1 — ELECTRONIC SUPPLEMENTARY MATERIAL [file 330_2025_11761_MOESM1_ESM.pdf]

Prognostic Value of [18F]fluorodeoxyglucose PET/CT in the new Staging System for Non-Small Cell Lung Cancer

ELECTRONIC SUPPLEMENTARY MATERIAL

Supplementary Tables

		AJCC/UICC 8th edition		AJCC/UICC 9th edition	
Variable	Categories	HR (95% CI)	*p*	HR (95% CI)	*p*
Age	<61				
	61-69	1.72 (1.38-2.13)	<0.001	1.69 (1.36-2.10)	<0.001
	>69	2.39 (1.91-2.98)	<0.001	2.37 (1.90-2.95)	<0.001
Sex	Female				
	Male	1.50 (1.23-1.82)	<0.001	1.51 (1.24-1.83)	<0.001
Treatment	Surgery[1]				
	Definitive RT[1]	2.76 (2.26-3.37)	<0.001	2.80 (2.29-3.42)	<0.001
	Neoadjuvant CCRT[2]	1.07 (0.84-1.35)	0.59	1.14 (0.91-1.44)	0.24
Type	Adenocarcinoma				
	Non-adenocarcinoma	1.09 (0.92-1.29)	0.34	1.08 (0.91-1.29)	0.37
Stage group	IA				
	IB	1.98 (1.49-2.63)	<0.001	1.99 (1.50-2.65)	<0.001
	IIA	2.43 (1.70-3.47)	<0.001	2.36 (1.72-3.24)	<0.001
	IIB	2.40 (1.83-3.13)	<0.001	2.58 (1.99-3.35)	<0.001
	IIIA	3.85 (2.99-4.95)	<0.001	4.21 (3.26-5.43)	<0.001
	IIIB+IIIC[3]	5.23 (3.89-8.03)	<0.001	4.44 (3.29-6.00)	<0.001
SUVmax	< 6.4				
	≥ 6.4	1.81 (1.47-2.24)	<0.001	1.78 (1.44-2.20)	<0.001

[1]With or without subsequent adjuvant chemotherapy, [2]followed by surgery. [3]Due to the small sample size of stage group IIIC, it was combined into the IIIB group. AJCC, American Joint Committee on Cancer; UICC, Union for International Cancer Control; HR, hazard ratio; CI, confidence interval; RT, radiotherapy; CCRT, concurrent chemoradiotherapy; SUVmax, maximum standardized uptake value.

Supplementary Table 1. Multivariate Cox regression analysis of overall survival in non-small cell lung cancer patients.

Variable	Categories	AJCC/UICC 8th edition HR (95% CI)	AJCC/UICC 8th edition *p*	AJCC/UICC 9th edition HR (95% CI)	AJCC/UICC 9th edition *p*
Age	<61				
	61-69	0.94 (0.79-1.11)	0.45	0.92 (0.78-1.09)	0.35
	>69	1.10 (0.91-1.33)	0.31	1.08 (0.90-1.30)	0.42
Sex	Female				
	Male	1.06 (0.91-1.25)	0.47	1.04 (0.89-1.22)	0.63
Treatment	Surgery[1]				
	Definitive RT[1]	0.99 (0.77-1.30)	0.92	1.00 (0.77-1.30)	0.99
	Neoadjuvant CCRT[2]	1.11 (0.91-1.38)	0.35	1.29 (1.05-1.59)	0.01
Type	Adenocarcinoma				
	Non-adenocarcinoma	0.59 (0.50-0.71)	<0.001	0.58 (0.49-0.69)	<0.001
Stage group	IA				
	IB	2.74 (1.71-2.68)	<0.001	2.76 (2.10-3.64)	<0.001
	IIA	3.50 (1.88-3.46)	<0.001	3.89 (2.85-5.32)	<0.001
	IIB	3.35 (2.11-3.28)	<0.001	3.99 (3.07-5.18)	<0.001
	IIIA	6.00 (4.75-7.92)	<0.001	5.57 (4.26-7.27)	<0.001
	IIIB+IIIC[3]	5.74 (4.20-8.19)	<0.001	5.11 (3.68-7.10)	<0.001
SUVmax	< 6.4				
	≥ 6.4	1.77 (1.47-2.13)	<0.001	1.84 (1.52-2.23)	<0.001

[1]With or without subsequent adjuvant chemotherapy, [2]followed by surgery. [3]Due to the small sample size of stage group IIIC, it was combined into the IIIB group. AJCC, American Joint Committee on Cancer; UICC, Union for International Cancer Control; HR, hazard ratio; CI, confidence interval; RT, radiotherapy; CCRT, concurrent chemoradiotherapy; SUVmax, maximum standardized uptake value.

Supplementary Table 2. Multivariate Cox regression analysis of disease-free survival in non-small cell lung cancer patients.

Stage group (9th)	Low SUVmax HR (95% CI) [n]	High SUVmax HR (95% CI) [n]
IA	1 [958]	5.42 (3.77-7.79) [235]
IB	1.89 (1.14-3.16) [194]	7.64 (5.34-10.92) [193]
IIA	4.07 (2.34-7.10) [75]	8.22 (5.54-12.18) [123]
IIB	3.38 (2.06-5.53) [121]	8.22 (5.96-11.34) [319]
IIIA	5.99 (3.81-9.44) [94]	11.16 (8.26-15.07) [416]
IIIB + IIIC	14.38 (7.90-26.18) [24]	12.17 (8.74-16.94) [220]

AJCC, American Joint Committee on Cancer; UICC, Union for International Cancer Control; SUVmax, maximum standardized uptake value; HR, hazard ratio; CI, confidence interval.

Supplementary Table 3. Hazard ratios of overall survival in each subgroup according to each American Joint Committee on Cancer (AJCC)/Union for International Cancer Control (UICC) 9th edition stage group and maximum standardized uptake value (SUVmax).

Variable	Categories	Overall Survival			Disease-free Survival		
		HR (95% CI)	*p*	Log-rank	HR (95% CI)	*p*	Log-rank
8th stage group	IA1			<0.001			<0.001
	IA2	1.28 (0.63-2.62)	0.50		1.33 (0.64-1.97)	0.68	
	IA3	1.77 (0.88-3.55)	0.11		1.84 (1.07-3.16)	0.03	
	IB	3.66 (1.84-7.28)	<0.001		3.64 (2.13-6.21)	<0.001	
	IIA	6.28 (3.06-12.88)	<0.001		5.29 (2.99-9.36)	<0.001	
	IIB	5.74 (2.91-11.29)	<0.001		5.28 (3.11-8.95)	<0.001	
	IIIA	6.97 (3.58-13.55)	<0.001		9.27 (5.54-15.52)	<0.001	
	IIIB+IIIC[1]	13.03 (6.62-25.66)	<0.001		12.54 (7.38-21.30)	<0.001	
9th stage group	IA1			<0.001			<0.001
	IA2	1.28 (0.63-2.62)	0.50		1.33 (0.64-1.97)	0.68	
	IA3	1.77 (0.88-3.55)	0.11		1.84 (1.07-3.16)	0.03	
	IB	3.66 (1.84-7.28)	<0.001		3.64 (2.13-6.20)	<0.001	
	IIA	5.34 (2.65-10.75)	<0.001		5.21 (3.01-9.00)	<0.001	
	IIB	5.63 (2.87-11.05)	<0.001		6.20 (3.67-10.45)	<0.001	
	IIIA	8.50 (4.36-16.57)	<0.001		9.64 (5.74-16.17)	<0.001	
	IIIB+IIIC[1]	10.54 (5.34-20.79)	<0.001		11.92 (7.02-20.21)	<0.001	

[1]Due to the small sample size of stage group IIIC, it was combined into the IIIB group. HR, hazard ratio; CI, confidence interval.

Supplementary Table 4. Univariate Cox regression analyses and log-rank analyses, considering the subgroups of stage group IA.

Variable	Categories	AJCC/UICC 8th edition		AJCC/UICC 9th edition	
		HR (95% CI)	*p*	HR (95% CI)	*p*
Age	<61				
	61-69	1.72 (1.38-2.13)	<0.001	1.69 (1.36-2.10)	<0.001
	>69	2.39 (1.91-2.98)	<0.001	2.37 (1.90-2.95)	<0.001
Sex	Female				
	Male	1.50 (1.23-1.82)	<0.001	1.51 (1.24-1.83)	<0.001
Treatment	Surgery[1]				
	Definitive RT[1]	2.76 (2.26-3.37)	<0.001	2.80 (2.29-3.42)	<0.001
	Neoadjuvant CCRT[2]	1.07 (0.84-1.35)	0.59	1.14 (0.91-1.44)	0.24
Type	Adenocarcinoma				
	Non-adenocarcinoma	1.09 (0.92-1.29)	0.34	1.08 (0.91-1.29)	0.37
Stage group	IA1				
	IA2	0.99 (0.49-2.04)	0.99	1.00 (0.49-2.04)	0.99
	IA3	0.99 (0.49-2.01)	0.98	1.00 (0.49-2.02)	0.99
	IB	1.97 (0.98-3.96)	0.06	1.99 (0.99-3.99)	0.054
	IIA	2.41 (1.16-5.02)	0.02	2.35 (1.15-4.80)	0.02
	IIB	2.39 (1.19-4.77)	0.01	2.57 (1.29-5.14)	0.01
	IIIA	3.82 (1.92-7.60)	<0.001	4.19 (2.10-8.36)	<0.001
	IIIB+IIIC[3]	5.19 (2.56-10.54)	<0.001	4.43 (2.17-9.00)	<0.001
SUVmax	< 6.4				
	≥ 6.4	1.81 (1.47-2.24)	<0.001	1.78 (1.44-2.20)	<0.001

[1]With or without subsequent adjuvant chemotherapy, [2]followed by surgery. [3]Due to the small sample size of stage group IIIC, it was combined into the IIIB group. AJCC, American Joint Committee on Cancer; UICC, Union for International Cancer Control; HR, hazard ratio; CI, confidence interval; RT, radiotherapy; CCRT, concurrent chemoradiotherapy; SUVmax, maximum standardized uptake value.

Supplementary Table 5. Multivariate Cox regression analysis of overall survival in non-small cell lung cancer patients, considering the subgroups of stage group IA.

Variable	Categories	AJCC/UICC 8th edition		AJCC/UICC 9th edition	
		HR (95% CI)	*p*	HR (95% CI)	*p*
Age	<61				
	61-69	0.94 (0.79-1.11)	0.45	0.92 (0.78-1.09)	0.35
	>69	1.10 (0.91-1.33)	0.31	1.08 (0.90-1.30)	0.42
Sex	Female				
	Male	1.06 (0.91-1.25)	0.47	1.04 (0.89-1.22)	0.63
Treatment	Surgery[1]				
	Definitive RT[1]	0.99 (0.77-1.30)	0.92	1.00 (0.77-1.30)	0.99
	Neoadjuvant CCRT[2]	1.11 (0.91-1.38)	0.35	1.29 (1.05-1.59)	0.01
Type	Adenocarcinoma				
	Non-adenocarcinoma	0.59 (0.50-0.71)	<0.001	0.58 (0.49-0.69)	<0.001
Stage group	IA1				
	IA2	1.02 (0.58-1.79)	0.94	1.02 (0.58-1.79)	0.93
	IA3	1.38 (0.80-2.37)	0.25	1.39 (0.81-2.40)	0.23
	IB	2.55 (1.48-4.37)	<0.001	2.76 (1.51-4.43)	<0.001
	IIA	3.04 (1.70-5.43)	<0.001	3.89 (1.87-5.68)	<0.001
	IIB	3.12 (1.82-5.36)	<0.001	3.99 (2.18-6.36)	<0.001
	IIIA	5.78 (3.39-9.84)	<0.001	5.57 (3.28-9.60)	<0.001
	IIIB+IIIC[3]	6.45 (3.70-11.23)	<0.001	5.11 (3.47-10.51)	<0.001
SUVmax	< 6.4				
	≥ 6.4	1.77 (1.47-2.13)	<0.001	1.84 (1.52-2.23)	<0.001

[1]With or without subsequent adjuvant chemotherapy, [2]followed by surgery. [3]Due to the small sample size of stage group IIIC, it was combined into the IIIB group. AJCC, American Joint Committee on Cancer; UICC, Union for International Cancer Control; HR, hazard ratio; CI, confidence interval; RT, radiotherapy; CCRT, concurrent chemoradiotherapy; SUVmax, maximum standardized uptake value.

Supplementary Table 6. Multivariate Cox regression analysis of disease-free survival in non-small cell lung cancer patients, considering the subgroups of stage group IA.

Variable	Categories	Overall Survival			Disease-free Survival		
		HR (95% CI)	*p*	Log-rank	HR (95% CI)	*p*	Log-rank
Age	<61			<0.001			<0.001
	61-69	2.30 (1.69-3.13)	<0.001		1.27 (1.03-1.55)	0.02	
	>69	4.09 (3.04-5.52)	<0.001		1.97 (1.61-2.41)	<0.001	
Age (cont.)		1.06 (1.05-1.08)	<0.001	<0.001	1.03 (1.02-1.04)	<0.001	<0.001
Sex	Female			<0.001			<0.001
	Male	2.45 (1.90-3.17)	<0.001		1.57 (1.32-1.86)	<0.001	
Cell type	Adenocarcinoma			<0.001			<0.001
	Non-adenocarcinoma	3.04 (2.43-3.81)	<0.001		1.87(1.56-2.23)	<0.001	
pT stage	T1a			<0.001			<0.001
	T1b	0.92 (0.44-1.91)	0.81		1.15 (0.64-2.05)	0.64	
	T1c	1.41 (0.70-2.85)	0.34		2.19 (1.26-3.81)	0.01	
	T2a	2.94 (1.48-5.84)	0.002		4.23 (2.46-7.30)	<0.001	
	T2b	4.74 (2.30-9.79)	<0.001		5.34 (2.99-9.56)	<0.001	
	T3	4.72 (2.33-9.56)	<0.001		5.25 (2.98-9.24)	<0.001	
	T4	8.21 (3.96-17.02)	<0.001		9.08 (5.02-16.40)	<0.001	
8th pN stage	N0			<0.001			<0.001
	N1	2.13 (1.55-2.95)	<0.001		2.75 (2.17-3.47)	<0.001	
	N2	2.90 (2.18-3.87)	<0.001		4.67 (3.81-5.73)	<0.001	
9th pN stage	N0			<0.001			<0.001
	N1	2.13 (1.55-2.95)	<0.001		2.75 (2.18-3.47)	<0.001	
	N2a	2.88 (2.10-3.96)	<0.001		4.50 (3.59-5.63)	<0.001	
	N2b	3.08 (1.76-5.39)	<0.001		5.67 (3.88-8.30)	<0.001	
8th pStage group	IA			<0.001			<0.001
	IB	3.07 (2.16-4.35)	<0.001		2.90 (2.24-3.74)	<0.001	
	IIA	4.65 (2.90-7.47)	<0.001		3.88 (2.67-5.62)	<0.001	
	IIB	3.85 (2.73-5.44)	<0.001		3.80 (2.95-4.88)	<0.001	
	IIIA	6.20 (4.50-8.54)	<0.001		7.23 (5.74-9.11)	<0.001	
	IIIB	11.41 (6.70-19.43)	<0.001		13.97 (9.22-21.16)	<0.001	
9th pStage group	IA			<0.001			<0.001
	IB	3.07 (2.16-4.35)	<0.001		2.90 (2.24-3.74)	<0.001	
	IIA	4.01 (2.66-6.04)	<0.001		4.04 (2.83-5.46)	<0.001	
	IIB	3.96 (2.80-5.60)	<0.001		4.02 (3.57-5.16)	<0.001	
	IIIA	7.59 (5.48-10.53)	<0.001		7.84 (5.62-9.99)	<0.001	
	IIIB	7.05 (3.89-12.77)	<0.001		12.00 (7.92-18.17)	<0.001	
SUVmax (categorized)	< 3.6			<0.001			<0.001
	≥ 3.6	7.02 (5.05-9.74)	<0.001		5.76 (4.63-7.16)	<0.001	
SUVmax (continuous)		1.10 (1.09-1.12)	<0.001	<0.001	1.09 (1.08-1.10)	<0.001	<0.001

HR, hazard ratio; CI, confidence interval; RT, radiotherapy; CCRT, concurrent chemoradiotherapy; SUVmax, maximum standardized uptake value.

Supplementary Table 7. Univariate Cox regression analyses and log-rank analyses of survival in non-small cell lung cancer patients with pathologic staging (pStage).

		Overall survival				Disease-free survival			
		AJCC/UICC 8th edition		AJCC/UICC 9th edition		AJCC/UICC 8th edition		AJCC/UICC 9th edition	
Variable	Categories	HR (95% CI)	*p*	HR (95% CI)	*p*	HR (95% CI)	*p*	HR (95% CI)	*p*
Age	<61								
	61-69	2.08 (1.52-2.85)	<0.001	1.97 (1.44-2.69)	<0.001	1.17 (0.95-1.44)	0.13	1.12 (0.91-1.38)	0.13
	>69	3.43 (2.53-4.66)	<0.001	3.41 (2.51-4.62)	<0.001	1.74 (1.42-2.13)	<0.001	1.71 (1.39-2.09)	<0.001
Sex	Female								
	Male	1.62 (1.22-2.14)	<0.001	1.65 (1.24-2.18)	<0.001	1.22 (1.01-1.48)	0.04	1.25 (1.03-1.51)	0.02
Type	Adenocarcinoma								
	Non-adenocarcinoma	1.18 (0.92-1.53)	0.20	1.16 (0.90-1.50)	0.25	0.81 (0.67-0.99)	0.04	0.78 (0.64-0.96)	0.02
Stage group	IA								
	IB	1.73 (1.21-2.49)	0.003	1.74 (1.21-2.50)	0.003	1.79 (1.37-2.34)	<0.001	1.80 (1.38-2.34)	<0.001
	IIA	2.30 (1.42-3.74)	<0.001	2.13 (1.40-3.25)	<0.001	2.21 (1.51-3.24)	<0.001	2.34 (1.71-3.20)	<0.001
	IIB	1.83 (1.27-2.64)	0.001	1.91 (1.32-2.75)	<0.001	2.03 (1.55-2.66)	<0.001	2.14 (1.64-2.80)	<0.001
	IIIA	3.54 (2.52-4.97)	<0.001	4.32 (3.05-6.13)	<0.001	4.11 (3.20-5.27)	<0.001	4.52 (3.48-5.86)	<0.001
	IIIB	6.06 (3.49-10.51)	<0.001	3.26 (1.77-6.00)	<0.001	6.83 (4.45-10.48)	<0.001	5.38 (3.50-8.27)	<0.001
SUVmax	< 3.6								
	≥ 3.6	3.57 (2.48-5.16)	<0.001	3.54 (2.45-5.12)	<0.001	3.53 (2.75-4.51)	<0.001	3.53 (2.76-4.52)	<0.001

AJCC, American Joint Committee on Cancer; UICC, Union for International Cancer Control; HR, hazard ratio; CI, confidence interval; RT, radiotherapy; CCRT, concurrent chemoradiotherapy; SUVmax, maximum standardized uptake value.

Supplementary Table 8. Multivariate Cox regression analysis in non-small cell lung cancer patients with pathologic staging (pStage).

Variable	Categories	Overall Survival			Disease-free Survival		
		HR (95% CI)	*p*	Log-rank	HR (95% CI)	*p*	Log-rank
Age	<61			<0.001			<0.001
	61-69	2.00 (1.56-2.57)	<0.001		1.21 (1.01-1.44)	0.04	
	>69	3.84 (3.05-4.84)	<0.001		1.82 (1.54-2.15)	<0.001	
Age (cont.)		1.06 (1.05-1.07)	<0.001	<0.001	1.02 (1.02-1.03)	<0.001	<0.001
Sex	Female			<0.001			<0.001
	Male	2.42 (1.97-2.98)	<0.001		1.73 (1.48-2.02)	<0.001	
Treatment	Surgery[1]			<0.001			<0.001
	Definitive RT[1]	6.42 (5.27-7.81)	<0.001		3.46 (2.91-4.12)	<0.001	
	Neoadjuvant CCRT[2]	3.25 (2.62-4.04)	<0.001		3.80 (3.23-4.47)	<0.001	
Cell type	Adenocarcinoma			<0.001			<0.001
	Non-adenocarcinoma	2.96 (2.50-3.51)	<0.001		1.76 (1.53-2.02)	<0.001	
T stage	T1a			<0.001			<0.001
	T1b	2.25 (0.90-5.60)	0.08		1.93 (0.97-3.83)	0.06	
	T1c	3.17 (1.29-7.80)	0.01		3.35 (1.72-6.55)	<0.001	
	T2a	5.07 (2.07-12.40)	<0.001		5.45 (2.79-10.61)	<0.001	
	T2b	8.49 (3.44-20.94)	<0.001		7.45 (3.78-14.69)	<0.001	
	T3	11.01 (4.51-26.92)	<0.001		8.71 (4.45-17.04)	<0.001	
	T4	11.61 (4.66-28.91)	<0.001		8.57 (4.29-17.14)	<0.001	
8th N stage	N0			<0.001			<0.001
	N1	2.31 (1.76-3.04)	<0.001		2.54 (2.02-3.19)	<0.001	
	N2	2.61 (2.17-3.13)	<0.001		3.84 (3.32-4.45)	<0.001	
	N3	7.16 (4.18-12.28)	<0.001		7.02 (4.31-11.43)	<0.001	
9th N stage	N0			<0.001			<0.001
	N1	2.31 (1.76-3.04)	<0.001		2.54 (2.02-3.19)	<0.001	
	N2a	2.50 (2.02-3.08)	<0.001		3.71 (3.14-4.38)	<0.001	
	N2b	2.86 (2.21-3.72)	<0.001		4.17 (3.40-5.11)	<0.001	
	N3	7.17 (4.18-12.29)	<0.001		7.03 (4.32-11.45)	<0.001	
8th stage group	IA			<0.001			<0.001
	IB	2.14 (1.54-2.96)	<0.001		2.49 (1.92-3.23)	<0.001	
	IIA	4.17 (2.81-6.20)	<0.001		3.87 (2.74-5.47)	<0.001	
	IIB	3.59 (2.68-4.81)	<0.001		3.66 (2.87-4.68)	<0.001	
	IIIA	4.33 (3.37-5.55)	<0.001		6.14 (5.03-7.50)	<0.001	
	IIIB+IIIC[3]	7.99 (5.99-10.67)	<0.001		8.51 (6.66-10.88)	<0.001	
9th stage group	IA			<0.001			<0.001
	IB	2.14 (1.54-2.96)	<0.001		2.49 (1.92-3.23)	<0.001	
	IIA	3.54 (2.49-5.02)	<0.001		3.83 (2.86-5.12)	<0.001	
	IIB	3.44 (2.59-4.56)	<0.001		4.26 (3.40-5.35)	<0.001	
	IIIA	5.30 (4.11-6.83)	<0.001		6.29 (5.11-7.76)	<0.001	
	IIIB+IIIC[3]	6.59 (4.90-8.85)	<0.001		8.24 (6.47-10.48)	<0.001	
SUVmax (categorized)	< 3.5			<0.001			<0.001
	≥ 3.5	9.02 (6.46-12.60)	<0.001		6.73 (5.35-8.47)	<0.001	
SUVmax (cont.)		1.09 (1.08-1.10)	<0.001	<0.001	1.08 (1.07-1.08)	<0.001	<0.001

[1]With or without subsequent adjuvant chemotherapy, [2]followed by surgery. [3]Due to the small sample size of stage group IIIC, it was combined into the IIIB group. HR, hazard ratio; CI, confidence interval; RT, radiotherapy; CCRT, concurrent chemoradiotherapy; SUVmax, maximum standardized uptake value.

Supplementary Table 9. Univariate Cox regression analyses and log-rank analyses of survival in non-small cell lung cancer patients originally diagnosed with the 7th edition of the American Joint Committee on Cancer (AJCC)/Union for International Cancer Control (UICC) staging system (diagnosed from Jan 2010 to Dec 2016).

Variable	Categories	Overall survival				Disease-free survival			
		AJCC/UICC 8th edition		AJCC/UICC 9th edition		AJCC/UICC 8th edition		AJCC/UICC 9th edition	
		HR (95% CI)	*p*	HR (95% CI)	*p*	HR (95% CI)	*p*	HR (95% CI)	*p*
Age	<61								
	61-69	1.64 (1.27-2.11)	<0.001	1.59 (1.24-2.05)	<0.001	1.01 (0.84-1.21)	0.90	0.98 (0.82-1.18)	0.86
	>69	2.32 (1.79-2.99)	<0.001	2.28 (1.77-2.95)	<0.001	1.37 (1.14-1.54)	<0.001	1.35 (1.12-1.62)	0.002
Sex	Female								
	Male	1.41 (1.13-1.77)	0.002	1.42 (1.14-1.78)	0.002	1.25 (1.06-1.47)	0.01	1.23 (1.05-1.46)	0.01
Treatment	Surgery[1]								
	Definitive RT[1]	2.77 (2.20-3.49)	<0.001	2.82 (2.24-3.55)	<0.001	1.93 (1.57-2.37)	<0.001	1.95 (1.59-2.39)	<0.001
	Neoadjuvant CCRT[2]	1.15 (0.88-1.50)	0.31	1.20 (0.92-1.55)	0.17	1.26 (1.03-1.55)	0.03	1.37 (1.12-1.67)	0.002
Type	Adenocarcinoma								
	Non-adenocarcinoma	1.06 (0.87-1.29)	0.54	1.06 (0.87-1.28)	0.59	0.74 (0.63-0.87)	<0.001	0.73 (0.62-0.85)	<0.001
Stage group	IA								
	IB	1.54 (1.11-2.15)	0.01	1.55 (1.11-2.16)	0.01	1.75 (1.34-2.29)	<0.001	1.75 (1.34-2.29)	<0.001
	IIA	2.15 (1.44-3.21)	<0.001	2.12 (1.49-3.03)	<0.001	2.25 (1.58-3.20)	<0.001	2.37 (1.76-3.20)	<0.001
	IIB	2.06 (1.53-2.79)	<0.001	2.10 (1.56-2.82)	<0.001	2.23 (1.73-2.89)	<0.001	2.48 (1.95-3.16)	<0.001
	IIIA	3.00 (2.26-3.99)	<0.001	3.35 (2.52-4.46)	<0.001	3.56 (2.81-4.52)	<0.001	3.47 (2.72-4.43)	<0.001
	IIIB + IIIC[3]	3.96 (2.85-5.48)	<0.001	3.45 (2.48-4.82)	<0.001	4.09 (3.07-5.45)	<0.001	3.91 (2.94-5.20)	<0.001
SUVmax	< 3.5								
	≥ 3.5	3.29 (2.26-4.78)	<0.001	3.27 (2.25-4.75)	<0.001	3.15 (2.42-4.10)	<0.001	3.17 (2.44-4.13)	<0.001

[1]With or without subsequent adjuvant chemotherapy, [2]followed by surgery. [3]Due to the small sample size of stage group IIIC, it was combined into the IIIB group. AJCC, American Joint Committee on Cancer; UICC, Union for International Cancer Control; HR, hazard ratio; CI, confidence interval; RT, radiotherapy; CCRT, concurrent chemoradiotherapy; SUVmax, maximum standardized uptake value.

Supplementary Table 10. Multivariate Cox regression analysis in non-small cell lung cancer patients originally diagnosed with the 7th edition of the American Joint Committee on Cancer (AJCC)/Union for International Cancer Control (UICC) staging system (diagnosed from Jan 2010 to Dec 2016).
